# Protein expression in *Pichia pastoris*: recent achievements and perspectives for heterologous protein production

**DOI:** 10.1007/s00253-014-5732-5

**Published:** 2014-04-18

**Authors:** Mudassar Ahmad, Melanie Hirz, Harald Pichler, Helmut Schwab

**Affiliations:** 1 Institute of Molecular Biotechnology, Graz University of Technology, Petersgasse 14/5, 8010 Graz, Austria; 2 Austrian Centre of Industrial Biotechnology (ACIB), Petersgasse 14, 8010 Graz, Austria

**Keywords:** Yeast, *Pichia pastoris*, Protein expression, Protein secretion, Protease-deficient strains, Chaperone

## Abstract

*Pichia pastoris* is an established protein expression host mainly applied for the production of biopharmaceuticals and industrial enzymes. This methylotrophic yeast is a distinguished production system for its growth to very high cell densities, for the available strong and tightly regulated promoters, and for the options to produce gram amounts of recombinant protein per litre of culture both intracellularly and in secretory fashion. However, not every protein of interest is produced in or secreted by *P. pastoris* to such high titres. Frequently, protein yields are clearly lower, particularly if complex proteins are expressed that are hetero-oligomers, membrane-attached or prone to proteolytic degradation. The last few years have been particularly fruitful because of numerous activities in improving the expression of such complex proteins with a focus on either protein engineering or on engineering the protein expression host *P. pastoris*. This review refers to established tools in protein expression in *P. pastoris* and highlights novel developments in the areas of expression vector design, host strain engineering and screening for high-level expression strains. Breakthroughs in membrane protein expression are discussed alongside numerous commercial applications of *P. pastoris* derived proteins.

## Introduction

The methylotrophic yeast *Pichia pastoris*, currently reclassified as *Komagataella pastoris*, has become a substantial workhorse for biotechnology, especially for heterologous protein production (Kurtzman 2009). It was introduced more than 40 years ago by Phillips Petroleum for commercial production of single cell protein (SCP) as animal feed additive based on a high cell density fermentation process utilizing methanol as carbon source. However, the oil crisis in 1973 increased the price for methanol drastically and made SCP production uneconomical. In the 1980s, *P. pastoris* was developed as a heterologous protein expression system using the strong and tightly regulated *AOX1* promoter (Cregg et al. 1985). In combination with the already developed fermentation process for SCP production, the *AOX1* promoter provided exceptionally high levels of heterologous proteins. One of the first large-scale industrial production processes established in the 1990s was the production of the plant-derived enzyme hydroxynitrile lyase at >20 g of recombinant protein per litre of culture volume (Hasslacher et al. 1997). This enzyme is used as biocatalyst for the production of enantiopure *m*-phenoxybenzaldehyde cyanohydrin — a building block of synthetic pyrethroids — on the multi-ton scale.

Through a far-sighted decision this expression system, initially patented by Phillips Petroleum, was made available to the scientific community for research purposes. A major breakthrough was the publication of detailed genome sequences of the original SCP production strain CBS7435 (Küberl et al. 2011), the first host strain developed for heterologous protein expression GS115 (De Schutter et al. 2009), as well as of the related *P. pastoris* DSMZ 70382 strain (Mattanovich et al. 2009b). Equally important breakthroughs for the commercial application of the *P. pastoris* cell factory were the Food and Drug Administration (FDA) GRAS (generally recognized as safe) status for a protein used in animal feed, phospholipase C (Ciofalo et al. 2006), and the FDA approval of a recombinant biopharmaceutical product, Kalbitor®, a kallikrein inhibitor (Thompson 2010).

The classical *P. pastoris* expression system has been extensively reviewed over the years (Cereghino and Cregg 2000; Daly and Hearn 2005; Gasser et al. 2013; Jin et al. 2006; Macauley-Patrick et al. 2005). In this review, we focus on recent developments for heterologous protein production and describe examples for the commercial use of this expression system. In the first chapter, we refer to the established basic vector systems and elaborate on developments thereof with an emphasis on newly developed promoter systems. Herein, also some aspects of secretion will be summarized. The second part is devoted to the most recent developments regarding host strain development. As a specific novelty, a new platform based on the CBS7435 strain is described, for which patent protection has ceased and no specific material rights are pending. In the third chapter, we describe specific strategies for obtaining high-level expression strains and summarize important applications of *P. pastoris* for production of biopharmaceuticals, membrane proteins and industrial proteins. The last section provides an outlook on future perspectives covering recent progress in molecular and cell biology of *P. pastoris* and possibilities for implementing new strategies in expression strain development.

## Basic systems for cloning and expression in *P. pastoris*

**Fig. 1:**
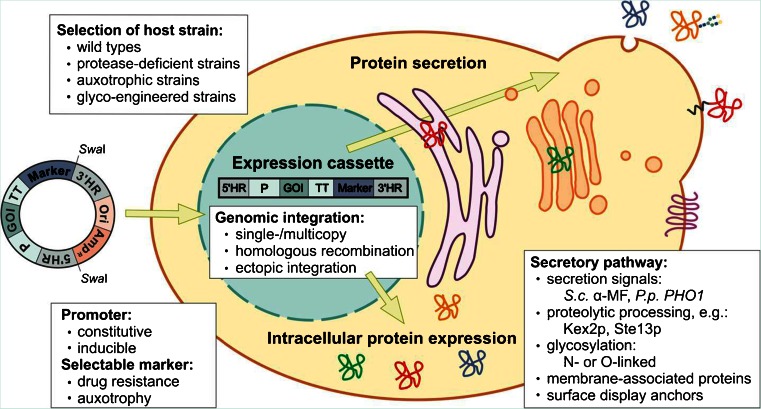
General considerations for heterologous gene expression in *P. pastoris*. Expression plasmids harbouring the gene(s) of interest (*GOI*) are linearized prior to transformation. Selectable markers (e.g., Amp^R^) and origin of replication (*Ori*) are required for plasmid propagation in *E. coli*. The expression level of the protein of interest may depend on (i) the chromosomal integration locus, which is targeted by the 5′ and 3′ homologous regions (5′HR and 3′HR), and (ii) on the gene copy number. A representative promoter (*P*) and transcription terminator (*TT*) pair are shown. Proper signal sequences will guide recombinant protein for intracellular or secretory expression, and will govern membrane integration or membrane anchoring

When devising strategies for cloning and expression of heterologous proteins in *P. pastoris* some points need to be considered from the start, that is, the choice of promoter–terminator combinations, suitable selection markers and application of vector systems for either intracellular or secreted expression including selection of proper secretion signals (Fig. 1). The choice of the proper expression vector and complementary host strain are a most important prerequisite for successful recombinant protein expression.

### Promoters

The use of tightly regulated promoters such as the alcohol oxidase (*AOX1*) promoter holds advantages for overexpression of proteins. By uncoupling the growth from the production phase, biomass is accumulated prior to protein expression. Therefore, cells are not stressed by the accumulation of recombinant protein during growth phase, and even the production of proteins that are toxic to *P. pastoris* is possible. Furthermore, it may be desirable to co-express helper proteins like chaperones at defined time points, for example, before the actual target protein is formed. On the other hand, use of constitutive promoters may ease process handling. Constitutive promoters are usually also applied to express selection markers. Metabolic pathway engineering strategies might further take advantage of fine-tuned constitutive promoters to ensure a controlled flux of metabolites. An extensive summary of promoters used for heterologous expression in *P. pastoris* has recently been published by Vogl and Glieder (2013). An overview of broadly used and extensively studied as well as recently examined promoters is given in Table 1.

**Table 1:** The most prominently used and very recently established promoters for heterologous expression in *P. pastoris*

Inducible	Corresponding gene	Regulation	Reference
*AOX1*	Alcohol oxidase 1	Inducible with MeOH	(Tschopp et al. 1987a)
*DAS*	Dihydroxyacetone synthase	Inducible with MeOH	(Ellis et al. 1985; Tschopp et al. 1987a)
*FLD1*	Formaldehyde dehydrogenase 1	Inducible with MeOH or methylamine	(Shen et al. 1998)
*ICL1*	Isocitrate lyase	Repressed by glucose, induction in absence of glucose/by addition of ethanol	(Menendez et al. 2003)
*PHO89*	Putative Na^+^/phosphate symporter	Induction upon phosphate starvation	(Ahn et al. 2009)
*THI11*	Thiamine biosynthesis gene	Repressed by thiamin	(Stadlmayr et al. 2010)
*ADH1*	Alcohol dehydrogenase	Repressed on glucose and methanol, induced on glycerol and ethanol	(Cregg and Tolstorukov 2012)
*ENO1*	Enolase	Repressed on glucose, methanol and ethanol, induced on glycerol	(Cregg and Tolstorukov 2012)
*GUT1*	Glycerol kinase	Repressed on methanol, induced on glucose, glycerol and ethanol	(Cregg and Tolstorukov 2012)
Constitutive	Corresponding gene	Regulation	Reference
*GAP*	Glyceraldehyde-3-P dehydrogenase	Constitutive expression on glucose, to a lesser extent on glycerol and methanol	(Waterham et al. 1997)
*TEF1*	Translation elongation factor 1	Constitutive expression on glycerol and glucose	(Ahn et al. 2007)
*PGK1*	3-Phosphoglycerate kinase	Constitutive expression on glucose, to a lesser extent on glycerol and methanol	(de Almeida et al. 2005)
*GCW14*	Potential glycosyl phosphatidyl inositol (GPI)-anchored protein	Constitutive expression on glycerol, glucose and methanol	(Liang et al. 2013b)
*G1*	High affinity glucose transporter	Repressed on glycerol, induced upon glucose limitation	(Prielhofer et al. 2013)
*G6*	Putative aldehyde dehydrogenase	Repressed on glycerol, induced upon glucose limitation	(Prielhofer et al. 2013)

#### Inducible promoters

The tightly regulated *AOX1* promoter (*P*_*AOX1*_), which was first employed for heterologous gene expression by Tschopp et al. (1987a), is still the most commonly used promoter (Lünsdorf et al. 2011; Sigoillot et al. 2012; Yu et al. 2013). *P*_*AOX1*_ is strongly repressed when *P. pastoris* is grown on glucose, glycerol or ethanol (Inan and Meagher 2001). Upon depletion of these carbon sources, the promoter is de-repressed, but is fully induced only upon addition of methanol. Several studies have identified multiple regulatory elements in the *P*_*AOX1*_ sequence (Hartner et al. 2008; Kranthi et al. 2006, 2009; Ohi et al. 1994; Parua et al. 2012; Staley et al. 2012; Xuan et al. 2009). Positively and negatively acting elements have been described (Kumar and Rangarajan 2012; Lin-Cereghino et al. 2006; Polupanov et al. 2012), but the molecular details of *P*_*AOX1*_ regulation are still not completely elucidated.

Methanol is a highly flammable and hazardous substance and, therefore, undesirable for large-scale fermentations. Alternative inducible promoters or *P*_*AOX1*_ variants, which can be induced without methanol but still reach high expression levels, are desired. A recently published patent application describes such a method, wherein expression is controlled by methanol-inducible promoters, such as *AOX1*, methanol oxidase (*MOX*) or formate dehydrogenase (*FMDH*), without the addition of methanol (Takagi et al. 2008). This was achieved by constitutively co-expressing the positively acting transcription factor Prm1p from either of the *GAP*, *TEF* or *PGK* promoters. The relative activity of a phytase reporter protein was 3-fold increased without addition of methanol as compared to a control strain with *PRM1* under its native promoter. However, phytase expression levels were not compared for standard methanol induction and constitutive Prm1p expression conditions. Hartner et al. have constructed a synthetic *AOX1* promoter library by deleting or duplicating transcription factor binding sites for fine-tuned expression in *P. pastoris* (Hartner et al. 2008). Using EGFP as reporter, some promoter variants were found to confer even higher expression levels than the native *P*_*AOX1*_ spanning a range between 6 % and 160 % of the native promoter activity. These *P*_*AOX1*_ variants have also proven to behave similarly when industrially relevant enzymes such as horseradish peroxidase and hydroxynitrile lyases were expressed.

Numerous further controllable promoters are currently being investigated for their ability to promote high-level expression (Table 1). For example, a recently published patent application describes the use of three novel inducible promoters from *P. pastoris*, *ADH1* (alcohol dehydrogenase), *GUT1* (glycerol kinase) and *ENO1* (enolase), showing interesting regulatory features (Cregg and Tolstorukov 2012). However, due to a lack of absolute expression values the performance of these novel promoters cannot be compared to the widely used *AOX1* and *GAP* promoters.

#### Constitutive promoters

Constitutive expression eases process handling, omits the use of potentially hazardous inducers and provides continuous transcription of the gene of interest. For this purpose, the glyceraldehyde-3-phosphate promoter (*P*_*GAP*_) is commonly used, which — on glucose — reaches almost the same expression levels as methanol-induced *P*_*AOX1*_ (Waterham et al. 1997). Expression levels from *P*_*GAP*_ drop to about one half on glycerol and to one third when cells are grown on methanol (Cereghino and Cregg 2000). Alternative constitutive promoters and promoter variants have been described recently (Table 1). The constitutive *P*_*GCW14*_ promoter, for example, was described to be a stronger promoter than the *GAP* and *TEF1* promoters, which was assessed by secretory expression of EGFP (Liang et al. 2013b). It was found that EGFP expression from *P*_*GCW14*_ yielded in a 10-fold increase compared to *P*_*GAP*_ driven expression when cells were cultivated on glycerol or methanol, and a 5-fold increase on glucose.

A recent DNA microarray study identified novel promoters that are repressed on glycerol, but are being induced upon shift to glucose-limited media (Prielhofer et al. 2013). Supposedly, the most interesting promoters discovered by this approach control expression of a high-affinity glucose transporter, *HGT1*, and of a putative aldehyde dehydrogenase. The former promoter was reported to drive EGFP expression to even higher levels than could be reached with *P*_*GAP*_. In glycerol fed-batch fermenter cultures, human serum album was expressed from the novel promoter to a 230 % increase in specific product yield as compared to *P*_*GAP*_ driven expression.

In some cases, it is desired that expression levels can be fine-tuned in order to (1) co-express accessory proteins facilitating recombinant protein expression and secretion or (2) provide protein post-translational modifications as well as to (3) engineer whole metabolic pathways consisting of a cascade of different enzymatic steps. For such applications, a library of *GAP* promoter variants with relative strengths ranging from 0.6 % to 16.9-fold of the wild type promoter activity was developed and tested using three different reporter proteins, yEGFP, β-galactosidase and methionine acetyltransferase (Qin et al. 2011).

### Vectors

The standard setup of vectors is a bi-functional system enabling replication in *E. coli* and maintenance in *P. pastoris* using as selection markers either auxotrophy markers (e.g., *HIS4*, *MET2*, *ADE1*, *ARG4*, *URA3*, *URA5*, *GUT1*) or genes conferring resistance to drugs such as Zeocin™, geneticin (G418) and blasticidin S. Although there are some reports of using episomal plasmids for heterologous protein expression or for the screening of mutant libraries in *P. pastoris* (Lee et al. 2005; Uchima and Arioka 2012), stable integration into the host genome is the most preferred method. Unlike in *Saccharomyces cerevisiae*, where homologous recombination (HR) predominates, non-homologous end-joining (NHEJ) is a frequent process in *P. pastoris.* The ratio of NHEJ and HR can be shifted towards HR by elongating the length of the homologous regions flanking the actual expression cassettes and by suppressing NHEJ efficiency (Näätsaari et al. 2012).

The standard vector systems for intracellular and secretory expression provided by Life Technologies (Carlsbad, CA, USA) include constitutive (*P*_*GAP*_) and inducible promoters triggered by methanol or methylamine (*P*_*AOX1*_, *P*_*FLD*_). The recently introduced PichiaPink™ expression kit for intracellular or secreted expression enables easy selection of multicopy integration clones by differences in colour formation based on *ade2* knockout strains and truncated *ADE2* promoters of varying strengths in front of the *ADE2* marker gene (Du et al. 2012; Nett 2010).

Additionally, BioGrammatics (Carlsbad, CA, USA) holds licences for selling standard *P. pastoris* expression vectors and strains and also provides GlycoSwitch® vectors for humanized glycosylation of target proteins (Table 2). Several vectors for disruption of *OCH1* and expression of different glycosidases or glycosyltransferases are available to achieve mammalian-type N-glycan structures in *P. pastoris*. These vectors harbour, for example, the human GlcNAc transferase I, the mannosidase II from rat, or the human galactosyl transferase I. A detailed protocol for humanizing the glycosylation pattern using the GlycoSwitch® vectors is provided (Jacobs et al. 2009).

**Table 2:** Commercial vector systems

Supplier	Promoter	Signal sequences	Selection in yeast	Selection in bacteria	Comments
Life Technologies™	*AOX1, FLD1,GAP*	*S. cerevisiae* α-MF; *P. pastoris PHO1*	Blasticidin, G418, Zeocin™, *HIS4*	Zeocin™, Ampicillin, Blasticidin	c-myc epitope, V5 epitope, C-terminal 6× His-tag available for detection/purification
Life Technologies –PichiaPink™	*AOX1*	α-MF; set of eight different signal sequences – not ready to use^a^	*ADE2*	Ampicillin	Low- and high-copy vectors available, *TRP2* sequence for targeting
BioGrammatics	*AOX1*	α-MF	Zeocin™, G418, Nourseothricin	Ampicillin	Intracellular or secreted expression
BioGrammatics – GlycoSwitch®	*GAP*	–	Zeocin™, G418, Hygromycin, *HIS4*, Nourseothricin	Zeocin™, Ampicillin, Kanamycin, Nurseothricin	Human GlcNAc transferase I, rat Mannosidase II, human Gal transferase I
DNA2.0	*AOX1*	Ten different signal sequences – ready to use^b^	Zeocin™, G418	Zeocin™, Ampicillin	Intracellular or secreted

^a^The different secretion signals have to be cloned into the vector by a three-way ligation step

^b^The α-MF secretion signal is provided once with Kex2p (KR) and Ste13p cleavage sites (EAEA), once lacking EA repeats, and once as truncated version (pre-region only)

James Cregg’s laboratory at the Keck Graduate Institute, Claremont, CA, USA, has developed a set of plasmids for protein secretion and intracellular expression in *P. pastoris* containing the strong *AOX1* promoter. These vectors are based on different auxotrophy markers, such as *ARG4*, *ADE1*, *URA3* and *HIS4*, for selection necessitating the use of the appropriate host strains (see section “Host strain development”). The vectors contain restriction sites for linearization within the marker genes to target the expression cassettes to the desired locus as well as for multicopy integration (Lin-Cereghino et al. 2001). Moreover, a set of integration vectors for sequential disruption of *ARG1*, *ARG2*, *ARG3*, *HIS1*, *HIS2*, *HIS5* and *HIS6* in *P. pastoris* was applied to provide the host strains for engineering the protein glycosylation pathway (Nett et al. 2005).

The Institute of Molecular Biotechnology, Graz University of Technology, Austria, provides vectors and strains to the *P. pastoris* community through the so-called ‘*Pichia* Pool’. The p*Pp* plasmids described by Näätsaari et al. (2012) comprise vectors containing the *GAP* or *AOX1* promoters and, for secretory expression, the *S. cerevisiae* α-mating factor (α-MF) secretion signal. The antibiotic selection marker cassettes were placed under the control of *ADH1* or *ILV5* promoters in the p*Pp*B1 and p*Pp*T4 vectors, respectively. It is described that the p*Pp*T4-based vectors usually lead to lower gene copies in the cell as compared to the p*Pp*B1-based vectors.

**Fig. 2:**
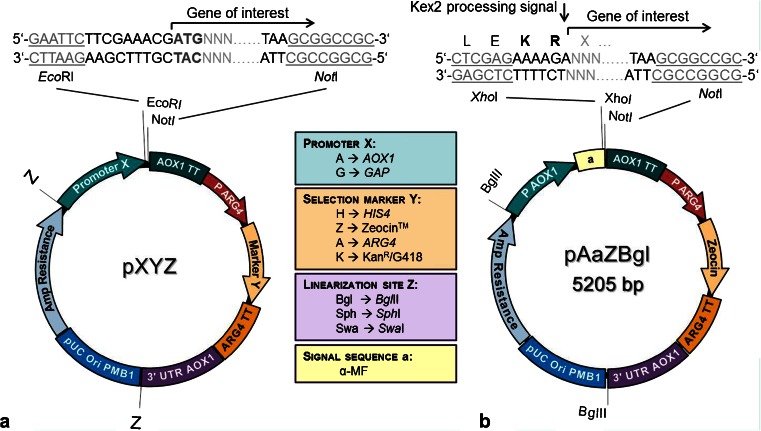
Novel ‘*Pichia* Pool’ plasmid sets for intracellular and secretory expression. **a** General features of pXYZ vector for intracellular expression. Letters refer to the choice of promoters (*X*), selection markers (*Y*), and restriction enzymes (*Z*) for linearization. Available elements are shown in boxes. The vector backbone harbours an ampicillin resistance marker and origin of replication for maintenance of the plasmid in *E. coli*. The GOI is *Eco*RI–*Not*I cloned directly after the promoter of choice. The Kozak consensus sequence for yeast (i.e., CGAAACG), should be restored between the *Eco*RI cloning site and the start codon of the GOI in order to achieve optimal translation. In addition, sequence variation within this region will allow fine-tuning translation initiation efficiency. Expression in *P. pastoris* is driven either by the methanol inducible *AOX1* or the constitutive *GAP* promoter. Positive clones can be selected for by antibiotic resistance (i.e., to Zeocin™ or geneticin sulphate) or by selection for His or Arg prototrophy. Selection marker expression is uniformly driven by the *ARG4* promoter–terminator pair. **b** Plasmid pAaZBgl from ‘*Pichia* Pool’ is shown as an example of a vector made for secretory expression encoding *S. cerevisiae* α-MF signal sequence in front of the GOI cloning site. The Kex2 processing site AAAAGA should be restored between the *Xho*I cloning site and the fusion point of the GOI

Further vectors based on either the *GAP* or the *AOX1* promoter and a series of strains have recently been added to this pool, both for intracellular and secretory protein expression (M. Ahmad, unpublished results). For intracellular expression, cloning of the target genes is accomplished by using *Eco*RI and *Not*I, whereby the Kozak consensus sequence has to be restored for efficient translation initiation (Fig. 2a). A special characteristic of these vectors is that the *Eco*RI site has been introduced by a single point mutation directly into the *AOX1* promoter sequence without changing the promoter activity. Thereby, the gene of interest may be fused to the promoter without having additional nucleotides between the promoter and the start codon. Another advantage is the use of the short *ARG4* promoter for the expression of the selection markers. The weaker *ARG4* promoter used for selection marker cassettes enables selection at lower concentrations of Zeocin™ (i.e., 25 instead of 100 μg/ml) without obtaining false-positive clones. For secretory expression governed by the *S. cerevisiae* α-MF signal sequence, *Xho*I and/or *Not*I sites are used for cloning the genes of interest (Fig. 2b).

### Aspects of secretory expression

One of the main advantages of using *P. pastoris* as a protein production host is its ability to secrete high titres of properly folded, post-translationally processed and active recombinant proteins into the culture media. As a rule of thumb, proteins secreted in their native hosts will also be secreted in *P. pastoris*. However, there are also some reports of successful secretion of typically intracellular proteins such as GFP or human catalase (Eiden-Plach et al. 2004; Shi et al. 2007). The most commonly employed secretion signals in *P. pastoris* are derived from *S. cerevisiae* α-MF, *S. cerevisiae* invertase (*SUC2*) and the *P. pastoris* endogenous acid phosphatase (*PHO1*) (Daly and Hearn 2005). As listed in Table 2, commercial kits also provide vectors with different secretion signals, which allows for screening of the best-suited signal sequence.

The α-MF signal sequence is composed of a pre- and pro-region and has proven to be most effective in directing protein through the secretory pathway in *P. pastoris*. The pre-region is responsible for directing the nascent protein post-translationally into the endoplasmic reticulum (ER) and is cleaved off subsequently by signal peptidase (Waters et al. 1988). The pro-region is thought to play a role in transferring the protein from ER to Golgi compartment and is finally cleaved at the dibasic KR site by the endo-protease Kex2p (Julius et al. 1984). The two EA repeats are subsequently trimmed by the *STE13* gene product (Brake et al. 1984). One of the common problems encountered while using the α-MF secretion signal is non-homogeneity of the N-termini of the recombinant proteins due to incomplete *STE13* processing. Constructs without the EA repeats may enhance homogeneity at the N termini of recombinant proteins. However, the removal of these sequences may affect protein yield. While no reports on enhanced co-expression of *STE13* are available, co-overexpression of *HAC1*, a transcription factor in the unfolded protein response (UPR) pathway, with the membrane protein adenosine A2 receptor had a positive effect on proper processing of the α-MF signal sequence (Guerfal et al. 2010). Recently, Yang et al. (2013) reported enhanced secretory protein production by optimizing the amino acid residues at the Kex2 P1′ site.

Multiple strategies have been followed to enhance the secretory potential of the α-MF signal sequence including codon optimization (Kjeldsen et al. 1998), directed evolution (Rakestraw et al. 2009), insertion of spacers and deletion mutagenesis (Lin-Cereghino et al. 2013). Directed evolution of the α-MF signal sequence in *S. cerevisiae* resulted in up to 16-fold enhanced full-length IgG_1_ secretion as compared to the wild type. Furthermore, when this improved leader sequence was combined with strain engineering strategies comprising *PDI* overexpression and elimination of proteins involved in vacuolar targeting, up to 180-fold enhanced secretion of the reporter protein was observed (Rakestraw et al. 2009). Deletion mutagenesis based on a predicted structure model of α-MF signal peptide resulted in 50 % increased secretion of horseradish peroxidase and *C. antarctica* lipase B (CALB) in *P. pastoris* (Lin-Cereghino et al. 2013). It appears that decreasing the hydrophobicity of the leader sequence by deleting hydrophobic residues or substituting them with more polar or charged residues increased the flexibility of the α-MF signal sequence structure, which enhanced the overall secretory capacity of the pro-region. Alternative signal sequences used to direct protein secretion and their features and applications are summarized in Table 3.

**Table 3:** Signal sequences used to secrete the protein into the extracellular space

Secretion signal	Source	Target protein(s)	Length	Reference
α-MF	*S.c.* α-mating factor	Most commonly used secretion signal in *P. pastoris*	85 aa, with or without EA repeats	(Brake et al. 1984)
PHO1	*P.p.* acid phosphatase	Mouse 5-HT5A, porcine pepsinogen,	15 aa	(Payne et al. 1995; Weiss et al. 1995; Yoshimasu et al. 2002)
SUC2	*S.c.* Invertase	Human interferon, α-amylase, α-1-antitrypsin	19 aa	(Moir and Dumais 1987; Paifer et al. 1994; Tschopp et al. 1987b)
PHA-E	Phytohemagglutinin	GNA, GFP and native protein	21 aa	(Raemaekers et al. 1999)
KILM1	Kl toxin	CM cellulase	44 aa	(Skipper et al. 1985)
pGKL	pGKL killer protein	Mouse α-amylase	20 aa	(Kato et al. 2001)
CLY and CLY-L8	C-lysozyme and syn. leucin-rich peptide	Human lysozyme	18 and 16 aa	(Oka et al. 1999)
K28 pre-pro-toxin	K28 virus toxin	Green fluorescent protein	36 aa	(Eiden-Plach et al. 2004)
Scw, Dse and Exg	*P.p.* Endogenous signal peptides	CALB and EGFP	19, 20 and 23 aa	(Liang et al. 2013a)
*Pp* Pir1	*P.p.* Pir1p	EGFP and Human α1-antitrypsin	61 aa	(Khasa et al. 2011)
HBFI and HBFII	Hydrophobins of *Trichoderma reesei*	EGFP	16 and 15 aa	(Kottmeier et al. 2011)

Beyond the choice of the secretion signals there are several other factors that govern efficient protein secretion. The newly synthesized proteins are translocated co- or post-translationally into the ER lumen through the Sec61p translocon. Then, proteins may undergo one or several posttranslational modifications, folding into the native state, disulphide-bond formation, glycosylation and membrane-anchoring. When the recombinant protein fails to fold into its native state or protein expression exceeds the folding capacity of the ER (Sha et al. 2013), unfolded proteins may start to aggregate, triggering the UPR pathway. UPR is responsible for induction of genes that are involved in protein folding. In parallel to UPR pathway, ER-associated degradation (ERAD) by the proteasome may relieve blocks in protein secretion (recently reviewed by Idiris et al. 2010 and Damasceno et al. 2012). Inappropriate mRNA structure and gene copy numbers, limits in transcription, translation and protein translocation into the ER, incomplete protein folding and inefficient protein targeting to the exterior of the cell are major bottlenecks encountered in secretory expression of heterologous proteins. Commonly used strategies to overcome such secretory bottlenecks comprise the overexpression of folding helper proteins like BiP/Kar2p, DnaJ, PDI, PPIs and Ero1p or, alternatively, overexpression of *HAC1*, a transcriptional regulator of the UPR pathway genes. Unlike in *S. cerevisiae*, Guerfal et al. (2010) reported that *HAC1* is constitutively expressed and spliced in *P. pastoris* under normal growth conditions, which may explain the higher titers of secreted proteins obtainable with this organism. A contradictory observation was reported by Whyteside et al. (2011). Un-spliced *HAC1* mRNA was detected under normal growth conditions and splicing of *HAC1* mRNA was only detected when cells were grown in presence of dithiothreitol (DTT) to activate the UPR. It should be mentioned, though, that sometimes overexpression of folding helpers actually reduced protein secretion or did not have any effect (van der Heide et al. 2002).

## Host strain development

Elucidation of full genome sequences and gene annotation were great steps toward rational strain engineering, identifying new promoters and progressing in the (systems) biology of *P. pastoris* (Küberl et al. 2011; Mattanovich et al. 2009a; De Schutter et al. 2009). Two online databases (http://bioinformatics.psb.ugent.be/orcae/overview/Picpa and http://www.pichiagenome.org) provide convenient access to genome sequences and annotations. Frequently used commercially available strains are the *his4* strain GS115, the reconstituted prototrophic strain X-33, the *aox1* knockout strains KM71 and KM71H as well as protease-deficient strains SMD1168 and SMD1168H and the *ade2* auxotrophic PichiaPink™ strain. Use of these strains for commercial applications, however, is restricted by patent protection and/or materials ownership policy. Strains derived from *P. pastoris* CBS7435, in contrast, are not covered by patent protection and, therefore represent an alternative for production purposes. Furthermore, the CBS7435 Mut^S^ strain provided by the Graz *Pichia* Pool has the advantage of being marker-free as it was constructed using the Flp/FRT recombinase system for marker removal (Näätsaari et al. 2012). Using the same strategy, *ade1* and *his4* knockout strains were created along with the CBS7435 *ku70* strain (CBS 12694), which is impaired in the NHEJ mechanism, thereby enhancing the efficiency of HR. A selection of most relevant strains is compiled in Table 4.

**Table 4:** *P. pastoris* host strains

Strain	Genotype	Phenotype	Source
Wild-type strains			
CBS7435 (NRRL Y-11430)	WT	WT	Centraalbureau voor Schimmelcultures, the Netherlands
CBS704 (DSMZ 70382)	WT	WT	Centraalbureau voor Schimmelcultures, the Netherlands
X-33	WT	WT	Life Technologies™
Auxotrophic strains			
GS115	*his4*	His^−^	Life Technologies™
PichiaPink™ 1	*ade2*	Ade^−^	Life Technologies™
KM71	*his4, aox1::ARG4, arg4*	His^−^, Mut^S^	Life Technologies™
KM71H	*aox1::ARG4, arg4*	Mut^S^	Life Technologies™
BG09	*arg4::nourseo* ^*R*^ *Δlys2::hyg* ^*R*^	Lys^−^, Arg^−^, Nourseothricin^R^, Hygromycin^R^	BioGrammatics
GS190	*arg4*	Arg^−^	(Cregg et al. 1998)
GS200	*arg4 his4*	His^−^, Arg^−^	(Waterham et al. 1996)
JC220	*ade1*	Ade^−^	(Cregg et al. 1998)
JC254	*ura3*	Ura^−^	(Cregg et al. 1998)
JC227	*ade1 arg4*	Ade^−^ Arg^−^	(Lin-Cereghino et al. 2001)
JC300-JC308	Combinations of *ade1 arg4 his4 ura3*	Combinations of Ade^−^, Arg^−^, His^−^, Ura^−^	(Lin-Cereghino et al. 2001)
YJN165	*ura5*	Ura^−^	(Nett and Gerngross 2003)
CBS7435 *his4* ^a^	*his4*	His^−^	(Näätsaari et al. 2012)
CBS7435 Mut^S^ *his4* ^a^	*aox1, his4*	Mut^S^, His^−^	(Näätsaari et al. 2012)
CBS7435 Mut^S^ *arg4* ^a^	*aox1, arg4*	Mut^S^, Arg^−^	(Näätsaari et al. 2012)
CBS7435 *met2* ^a^	*met2*	Met^−^	(*Pp*7030)^b^
CBS7435 *met2 arg4* ^a^	*met2 arg4*	Met^−^ Arg^−^	(*Pp*7031)^b^
CBS7435 *met2 his4* ^a^	*met2 his4*	Met^−^ His^−^	(*Pp*7032)^b^
CBS7435 *lys2* ^a^	*lys2*	Lys^−^	(*Pp*7033)^b^
CBS7435 *lys2 arg4* ^a^	*lys2 arg4*	Lys^−^ Arg^−^	(*Pp*7034)^b^
CBS7435 *lys2 his4* ^a^	*lys2 his4*	Lys^−^ His^−^	(*Pp*7035)^b^
CBS7435 *pro3* ^a^	*pro3*	Pro^−^	(*Pp*7036)^b^
CBS7435 *tyr1* ^a^	*tyr1*	Tyr^−^	(*Pp*7037)^b^
Protease-deficient strains			
SMD1163	*his4 pep4 prb1*	His^−^	(Gleeson et al. 1998)
SMD1165	*his4 prb1*	His^−^	(Gleeson et al. 1998)
SMD1168	*his4 pep4::URA3 ura3*	His^−^	Life Technologies™
SMD1168H	*pep4*		Life Technologies™
SMD1168 kex1::SUC2	*pep4::URA3 kex1::SUC2 his4 ura3*	His^−^	(Boehm et al. 1999)
PichiaPink 2-4	Combinations of *prb1/pep4*	Ade^−^	Life Technologies™
BG21	*sub2*		BioGrammatics
CBS7435 *prc1* ^a^	*prc1*		(*Pp*6676)^b^
CBS7435 *sub2* ^a^	*sub2*		(*Pp*6668)^b^
CBS7435 *sub2* ^a^	*his4 pep4*	His^−^	(*Pp*6911)^b^
CBS7435 *prb1* ^a^	*prb1*		(*Pp*6912)^b^
CBS7435 *his4 pep4 prb1*	*his4 pep4 prb1*	His^−^	(*Pp*7013)^b^
Glyco-engineered strains			
SuperMan_5_	*his4 och1::pGAPTrα1,2-mannosidase*	His^−^, Blasticidin^R^	BioGrammatics
	*och1::pGAPTrα1,2-mannosidase*	Blasticidin^R^	BioGrammatics
	*pep4 och1::pGAPTrα1,2-mannosidase*	Blasticidin^R^	BioGrammatics
Other strains			
GS241	*fld1*	Growth defect on methanol as sole C-source or methylamine as sole N-source	(Shen et al. 1998)
MS105	*his4 fld1*	See GS241; His^−^	(Shen et al. 1998)
MC100-3	*his4 arg4 aox1::ScARG4 aox2::PpHIS4*	Mut^−^	(Cregg et al. 1989)
CBS7435 *ku70* ^a^	*ku70*	WT	(Näätsaari et al. 2012)
CBS7435 *ku70 his4* ^a^	*ku70, his4*	His^−^	(Näätsaari et al. 2012)
CBS7435 *ku70 gut1*	*ku70, gut1*	Growth defect on glycerol; Zeocin^R^	(Näätsaari et al. 2012)
CBS7435 *ku70 ade1*	*ku70, ade1*	Ade^−^, Zeocin^R^	(Näätsaari et al. 2012)

^a^These *P. pastoris* CBS7435 derived strains are marker-free knockouts

^b^Strains from ‘*Pichia* Pool’ of TU Graz (M. Ahmad, unpublished results)

### Auxotrophic strains

Several auxotrophic strains (e.g., *ade1*, *arg4*, *his4*, *ura3*, *met2*), and combinations thereof are available together with vectors harbouring the respective genes as selectable markers (Lin-Cereghino et al. 2001; Thor et al. 2005, Graz *Pichia* Pool). Auxotrophic strains have been useful for in vivo labelling of proteins, for example in the global fluorination of *Candida antarctica* lipase B (CALB) in a *P. pastoris* X-33 *aro1* strain deficient in tryptophan, tyrosine, and phenylalanine biosynthesis (Budisa et al. 2010). Fluorinated analogues of these amino acids were supplemented and incorporated into the heterologous protein, thereby, for example, prolonging CALB shelf-life but lowering its lipase activity. The proteolytic pattern of CALB was retained, though. Another example is the use of a *lys2 arg4* double knockout strain for stable isotope labelling by amino acids in cell culture (SILAC) (Austin et al. 2011).

### Protease-deficient strains

Undesired proteolysis of heterologous proteins expressed in *P. pastoris* does not only lower the product yield or biological activity, but also complicates downstream processing of the intact product as the degradation products will have similar physicochemical and affinity properties. Proteolysis may occur either during vesicular transport of recombinant protein by secretory pathway-resident proteases (Werten and de Wolf 2005; Ni et al. 2008) or in the extracellular space by proteases being secreted, cell wall-associated (Kang et al. 2000) or released into the culture medium as a result of cell disruption during high cell density cultivation (Sinha et al. 2005). Different strategies have been employed to address the proteolysis problem, namely, modifying fermentation parameters (pH, temperature and specific growth rate), changing the media composition (rich medium, addition of casamino acids or peptone as competing substrates), lowering the salt concentration and addition of soytone (Zhao et al. 2008), applying protein engineering strategies (Gustavsson et al. 2001) and engineering of the expression host to obtain protease-deficient strains (reviewed by Idiris et al. 2010 and Macauley-Patrick et al. 2005). However, in some cases, optimization of the fermentation media and protein engineering strategies failed to alleviate the proteolysis problem and tuning the expression host itself was the only viable option (Li et al. 2010). The use of protease-deficient strains such as SMD1163 (*Δhis4 Δpep4 Δprb1*), SMD1165 (*Δhis4 Δprb1*) and SMD1168 (*Δhis4 Δpep4*) has been well documented for the expression of protease-sensitive proteins (Gleeson et al. 1998). *PEP4* encodes a major vacuolar aspartyl protease which is able to activate itself as well as further proteases such as carboxypeptidase Y (*PRC1*) and proteinase B (*PRB1*). The use of protease-deficient strains other than the above mentioned (e.g., *yps1*, *kex1*, *kex2*) was reported with variable success (Ni et al. 2008; Werten and de Wolf 2005; Wu et al. 2013; Yao et al. 2009). A general conclusion from these studies is that in many cases several proteases are involved in degradation events and, therefore, it is not an easy task to optimize protein expression by knocking out just a single one. However, the *pep4* and *prb1* knockout strains are still the most effective ones in preventing recombinant protein degradation, and, hence, also the most widely applied. Although it has been reported that protease-deficient strains show typically slower growth rates, lower transformation efficiencies and reduced viability (Lin-Cereghino and Lin-Cereghino 2007), experiments in our laboratory showed robust growth behaviour of 28 protease-deficient strains that were recently created (M. Ahmad, unpublished results).

### Glyco-engineered strains

When yeasts such as *P. pastoris* are chosen for production of therapeutic proteins, N- and O-linked glycosylation are of tremendous relevance. Although the assembly of the core glycans, that is, (Man)_8_-(GlcNAc)_2_, in the ER is highly conserved in mammals and yeasts, mammals provide a much higher diversity in the ultimate glycan structure assembled in the Golgi cisternae. Yeasts, in contrast, produce high mannose glycan structures, which may lead to decreased serum half-life and may trigger allergic reactions in the human body (Ballou 1990). While in *P. pastoris* the hyper-mannosylation is not as prominent as in *S. cerevisiae*, it is still a problem that needs to be tackled, and is therefore a target for intensive strain engineering. A very detailed summary of the glycosylation machinery and the targets for glyco-engineering in different yeast species, including *P. pastoris*, has been given recently (De Pourcq et al. 2010). To sum up briefly, engineering strategies included the introduction of a *Trichoderma reesei* α-1,2-mannosidase (Callewaert et al. 2001), the knockout of the highly conserved yeast Golgi protein α-1,6-mannosyltransferase encoded by *OCH1*, which is responsible for hyperglycosylation (Choi et al. 2003; Vervecken et al. 2004), as well as co-overexpression of several glycosyltransferases and glycosidases carrying proper targeting signals (Hamilton et al. 2003). Terminally sialylated glycoproteins produced for the first in *P. pastoris* were obtained by introducing a complex sialic acid pathway (Hamilton et al. 2006). Key to success was the correct localization of the heterologous glycosyltransferases and glycosidases in the ER and Golgi networks. Combinatorial genetic libraries and high throughput screening methods were successfully applied to find the best targeting signal/enzyme combinations for N-linked glycoengineering (Nett et al. 2011). Furthermore, a useful guide to glyco-engineering in *P. pastoris* by using the GlycoSwitch® technology was described by Jacobs et al. (2009). These strategies, altogether, enable the production of valuable biopharmaceuticals with a more homogeneous, ‘humanized’ N-glycosylation pattern.

However, as yeasts also carry out O-glycosylation that differs structurally from the mammalian type (Strahl-Bolsinger et al. 1999), O-glycosylation has also been an interesting target for engineering. In *P. pastoris*, O-linked glycosylation is initiated with a mannose monosaccharide, which is further elongated by α-1,2-mannose residues and finally capped with β- or phospho-mannose residues. Until lately, the engineering strategies were limited to the use of an inhibitor of the major ER located protein-O-mannosyltransferases (PMTs) as the deletion of these genes did not yield robust and viable strains. The characterization of the *P. pastoris* PMT gene family was an important step forward in O-glycosylation engineering (Nett et al. 2013). In this study, the knockout of PMTs as well as the use of PMT inhibitors led to a reduced number of O-mannosylation events and, furthermore, to reduced chain lengths of the O-glycans. A follow-up study described the production of a TNFR2:Fc^1^ fusion protein carrying sialylated O-linked glycans in *P. pastoris* (Hamilton et al. 2013). Therein, an α-1,2-mannosidase as well as a protein-O-linked-mannose β-1,2-N-acetylglucosaminyl-transferase 1 (PomGnT1) were co-expressed in a *P. pastoris* strain, that was already engineered in its N-glycosylation pathway. Hence, the mannose residues were first trimmed to single O-linked mannose residues, which were then capped with N-acetylglucosamine. This structure was extended with sialic acid residues to achieve human-like O-glycan residues similar to the α-dystroglycan-type. However, there is still room for improvement, for example by engineering *P. pastoris* towards human mucin-type O-glycosylation.

## Expression strategies and industrial applications

### Screening for high level expression

Subsequent to the choice of suitable expression vectors and proper host strains, and transformation of the expression cassettes, it is important to select for transformants which show high expression levels of the desired protein. Single copy transformants can be easily generated by targeting the linear expression cassettes to the *AOX1* locus resulting in gene replacement events. Ectopic integrations may simultaneously occur, however. Transformants resulting from gene replacement at the *AOX1* locus have methanol utilization slow phenotype (Mut^S^) and can be easily identified by replica-plating on minimal methanol plates. The most commonly applied strategy to screen for high-yielding *P. pastoris* transformants focusses on screening for clones having multicopy integrations of the expression cassette. A recent detailed review describes the methods applied to obtain strains containing multiple expression cassettes and provides a summary of published data showing correlations between copy number and expression levels of intracellular as well as secreted proteins. It also highlights the problem of genetic instability of the integration cassettes that might be encountered when cultivating multicopy strains. Due to the highly recombinogenic nature of *P. pastoris*, expression cassettes might be excised through loop-out recombination. This effect seems to be more pronounced the more copies are integrated (Aw and Polizzi 2013).

Regarding the correlation between copy number and expression level, a number of recent studies have shown a direct correlation especially for intracellular expression (Marx et al. 2009; Vassileva et al. 2001). The direct correlation of expression level and gene copy number is, however, not necessarily valid when the protein is directed to the secretory pathway. The most commonly employed method of generating multicopy expression strains in *P. pastoris* is based on plating the transformation mixture directly on selection plates containing increasing concentrations of antibiotics (e.g., 100 to 2,000 μg/ml of Zeocin™). The majority of transformants will have a single copy of the expression vector integrated into the genome, and numerous clones will have to be screened to find high-copy transformants (Lin-Cereghino and Lin-Cereghino 2007). Therefore, several high-throughput methods have been established to screen a large number of clones based on small-scale cultivation in deep well plates (Mellitzer et al. 2012; Weinhandl et al. 2012; Weis et al. 2004). The selected clones, however, might not perform as well in fermenter cultivations due to different cultivation conditions. A further pronounced problem of resistance marker based screening is a high prevalence of false-positive colonies. This so-called high transformation background is supposedly caused by cell stress and cell rupture. Depending on the mechanism of antibiotic resistance conferred by the resistance marker, un-transformed cells may survive in the vicinity of ruptured transformants. This problem was addressed by constructing expression vectors based on marker gene expression driven by the weak *ARG4* promoter (*Pichia* Pool, Fig. 2). This ensures basal levels of expression, thereby allowing handlers to select single copy to multicopy strains by plating the transformants directly on low concentrations of Zeocin™ (i.e., 25 μg/ml for single copy and up to 400 μg/ml for multi-copy transformants). Thus, transformants having 1 to 20 (±5) copies can be selected. To reduce the chances of having single copy transformants, regeneration time should be kept short and transformants should be plated directly on increased concentrations of antibiotic. By employing this method, only few transformants survive on high concentrations of antibiotic, but will most likely contain multiple copies, which can be determined by quantitative (qPCR) or Southern blot analysis (M. Ahmad, unpublished results). Performance can then be tested directly under production conditions in bioreactor cultivations instead of small-scale cultivations in deep well plates or shake flasks.

### Membrane protein expression

*P. pastoris* has been shown to produce 15+ g of soluble recombinant protein per litre of culture intracellularly (Hasslacher et al. 1997) or in secretory mode (Werten et al. 1999). Key to such high titres is the ability of *P. pastoris* to grow to very high cell densities reaching up to 150 g cell dry weight per litre of fermentation broth in fed-batch bioreactor cultivations (Jahic et al. 2006). At very high cell densities, even proteins that are present in limited entities per single cell can be produced with reasonable volumetric yields in *P. pastoris*. Typical examples of non-abundant proteins with high scientific and commercial relevance are integral membrane proteins. Being the targets of >50 % of drugs applied on humans (Arinaminpathy et al. 2009), only very few membrane proteins have been characterized on the molecular level regarding structure–function relationships. The simple reason is that it is difficult to obtain sufficient purified membrane protein for structural and biochemical studies, unless affinity-tagged membrane proteins are obtained at reasonable yield. Actually, *P. pastoris* has been applied routinely to produce affinity-tagged membrane proteins for protein purification and subsequent biochemical studies (Cohen et al. 2005; Haviv et al. 2007; Lifshitz et al. 2007). Furthermore, *P. pastoris* has been the expression host of choice for elucidating the crystal structures of membrane proteins from diverse origins, even from higher eukaryotes (Brohawn et al. 2012; Hino et al. 2012; Ho et al. 2009).

Evolutionary proximity of a heterologous expression host and the origin of an expressed membrane protein are beneficial for successful recombinant expression (Grisshammer and Tateu 2009). In addition to the intramolecular forces and bonds, ions, cofactors and interacting proteins that stabilize soluble proteins, membrane proteins are usually interacting with and are partially also stabilized by the lipids of the surrounding bilayers (Adamian et al. 2011). As *P. pastoris* and other yeast expression hosts do significantly differ in their membrane compositions from bacterial, plant or animal cells (Wriessnegger et al. 2007, 2009; Zinser and Daum 1995), heterologous membrane proteins may face stability issues upon expression in distantly related hosts. Thus, multiple approaches have been undertaken to improve *P. pastoris* host strains and expression conditions for membrane protein production. Applying similar tools as for the optimisation of soluble protein expression — that is, manipulation of expression conditions, addition of chemical chaperones, co-expression of chaperones or of proteins activating UPR, use of protease deficient strains, etc. — has been showing some, however often target-specific success in membrane protein expression. A novel approach is the engineering of *P. pastoris* cellular membranes for improved accommodation of heterologous membrane proteins. In the first reported example, a cholesterol-producing *P. pastoris* strain was shown to stably express an enhanced level of ligand-binding human Na,K-ATPase moieties on the cell surface (Hirz et al. 2013).

### Products on — or on the way to — the market

The *P.* pastoris expression system has gained importance for industrial application as highlighted by the number of patents published on heterologous expression in and cell engineering of *P. pastoris* (Bollok et al. 2009). Products obtained by heterologous expression in *P. pastoris* have already found their way to the market, as FDA approved biopharmaceuticals or industrial enzymes have shown. The www.pichia.com web page provides a list of proteins produced in *P. pastoris* with the commercial expression system licensed by Research Corporation Technologies (RCT) and their applications: Phytase (Phytex, Sheridan, IN, USA) is applied as animal feed additive to cleave plant derived phytate, thereby providing a source of phosphate. Trypsin (Roche Applied Science, Germany) is used, for example, as protease in proteomics research to obtain peptide patterns for MS analysis. Further examples listed are nitrate reductase (The Nitrate Elimination Co., Lake Linden, MI, USA), used for water testing and treatment, phospholipase C (Verenium, San Diego, CA, USA/DSM, The Netherlands), used for degumming of vegetable oils, and Collagen (Fibrogen, San Francisco, CA, USA), used in medical research and as dermal filler. Thermo Scientific (Waltham, MA, USA) sells recombinant *Tritirachium album* Proteinase K produced in *P. pastoris*. Concerning biopharmaceuticals, a famous example is Kalbitor® (ecallantide), produced in *P. pastoris* by Dyax (Cambridge, MA, USA). Kalbitor® is a plasma kallikrein inhibitor indicated against hereditary angioedema. This product was the first biopharmaceutical to be approved by the FDA for market release in 2009 (Walsh 2010). As can be found on the web page of RCT (www.rctech.com), *Pichia*-manufactured Jetrea®, a drug used for treatment of symptomatic vitreomacular adhesion, was recently approved by the FDA and the European Commission. Other *Pichia*-derived products provided by the Indian company Biocon are recombinant human insulin and analogues thereof (Insulin, Glargine). Products under development, such as Elastase inhibitor against Cystic fibrosis or Nanobody® ALX antibody fragments developed by Ablynx (Belgium), are also listed by Gerngross (2004) and on www.pichia.com. In 2008, Novozymes (Denmark), which found a highly active antimicrobial agent, the plectasin peptide derivative NZ2114 (Andes et al. 2009; Mygind et al. 2005), granted Sanofi-Aventis (France) an exclusive licence for the production and commercialisation of this compound in *P. pastoris.* This might be the first antimicrobial peptide approved for the market in the future.

Although not yet approved for medical use, many products can be found on the market for research purposes. GenScript (Piscataway, NJ, USA) provides recombinant cytokines and growth factors, such as human HSA-IFN-Alpha 2b, human Stem Cell Factor SCF, murine TNF-α and ovine IFN-τ, to name just a few examples. Recombinant human angiostatin can be found for instance in the reagents offered by Sigma-Aldrich (St. Louis, MO, USA).

## Future perspectives — outlook

Successful expression of many industrial enzymes as well as pharmaceutically relevant proteins has rendered the methylotrophic yeast *P. pastoris* one of the most suitable and powerful protein production host systems. It is also an emerging host for the expression of membrane proteins (Hirz et al. 2013) and of small bioactive and antimicrobial peptides, which could be a forthcoming alternative to chemical synthesis (Zhang et al. 2014). Although many basic elements of this expression system are now well developed and one can make use of a broad variety of vectors and host strains, there is still space for further optimization of protein expression and secretion, which, in many cases, will be highly dependent on the desired product. One general interest is to find effective alternatives for induction to replace methanol for industrial scale fermentations (Delic et al. 2013; Prielhofer et al. 2013; Stadlmayr et al. 2010).

Improving protein secretion performance is one of the first and foremost goals for engineering *P. pastoris*. There is still potential to increase yields, for example, by employing different secretion signals (Vadhana et al. 2013) or mutating *S. cerevisiae* α-MF (Lin-Cereghino et al. 2013). In contrast to the well-studied secretory pathway of *S. cerevisiae*, *P. pastoris* still is a black box regarding factors influencing secretion efficiency. Current studies try to identify these factors by mutagenesis approaches and screening for enhanced secretion of reporter proteins (Larsen et al. 2013; C. Winkler and H. Pichler, unpublished results). The well-developed tools for strain engineering, including marker-free integration and deletion of desired genes, will provide a powerful set of engineered designer host strains in the near future. These will provide optimized cell factories by fine-tuned co-expression of important homologous or heterologous protein functions needed for efficient and accurate functional expression, secretion and post-translational modification of proteins. Moreover, knockout or knockdown of undesired functions such as proteolytic decay will increase product quality and process performance. Considering the scope of this review on heterologous protein expression, it was not feasible to address all possible applications for *P. pastoris* as production organism, such as metabolic engineering for production of small molecules and metabolites, or for whole-cell biocatalysis. However, developments in these fields may also be relevant for constructing improved host strains dedicated for protein production. There are several recent reviews and research articles describing advances in these fields in detail (Abad et al. 2010; Araya-Garay et al. 2012; Wriessnegger and Pichler 2013).

## Footnotes

1 Ectodomain of tumor necrosis factor 2 with crystallizable fragment of IgG1 (Fc)
